# From HPV Detection to Cancer Prevention: The Role of Infection Persistence, Genotype, and Anatomical Site in HPV-Associated Cancers

**DOI:** 10.3390/jcm15208098

**Published:** 2026-10-19

**Authors:** Sara Jouya, Zahra Shahabinia, Mai Abdel Haleem A. Abusalah, Afrooz Mazidimoradi, Leila Allahqoli, Hamid Salehiniya, Do-Youn Lee

**Affiliations:** 1 Health Deputy, Birjand University of Medical Sciences, Birjand 9717853577, Iran; 2 Geriatric Health Research Center, Birjand University of Medical Sciences, Birjand 9717853577, Iran; 3 Department of Medical Laboratory Sciences, Faculty of Allied Medical Sciences, Al-Ahliyya Amman University, Amman 19328, Jordan; 4 Shiraz Health Assistant Department, Shiraz University of Medical Sciences, Shiraz 7193711351, Iran; 5 Faculty of Health Sciences, Cyprus International University, Nicosia 99258, Cyprus; 6 Department of Epidemiology and Biostatistics, School of Health, Social Determinants of Health Research Center, Birjand University of Medical Sciences, Birjand 9717853577, Iran; 7 College of General Education, Kookmin University, Seoul 02707, Republic of Korea

**Keywords:** human papillomavirus, cancer prevention, infection persistence, genotype, anatomical site

## Abstract

**Background/Objectives:** Most HPV infections are transient, but persistent high-risk infection, particularly with HPV16, is among the most important determinants of progression to HPV-associated precancer and cancer. The role of genotype, infection persistence, and anatomical site varies across cervical, oropharyngeal, anal, vaginal, vulvar, and penile cancers. This review synthesizes evidence to clarify how these factors shape cancer risk and prevention. **Methods:** This structured narrative review used iterative, topic-specific searches in PubMed, Scopus, Web of Science, and Google Scholar without a publication-year restriction and was limited to English-language sources. Search terms were adapted to each database and thematic question. Original empirical studies and secondary data analyses informed direct quantitative discussion, whereas reviews, meta-analyses, guidelines, consensus statements, and authoritative reports were used principally for context. Evidence was synthesized qualitatively because study designs, populations, anatomical sites, assays, outcomes, and clinical purposes differed substantially; no new meta-analysis was performed. **Results:** The most developed longitudinal and screening evidence concerned cervical cancer, whereas evidence for several non-cervical sites was smaller and more heterogeneous. HPV16 was the most important genotype in most cancers examined. The role of HPV18 and other high-risk genotypes varied depending on the cancer site, tissue type, and geographic region. Most transient infections do not lead to cancer; however, persistent high-risk HPV infection, particularly HPV16, was associated with high-grade lesions and an increased risk of cancer. Vaccination was associated with a reduction in high-grade lesions and invasive cancer in population-based studies. Self-sampling and urine testing had practical value when individuals with positive results proceeded to triage, treatment, and follow-up. **Conclusions:** Three key questions exist for assessing the risk of HPV-associated cancers: Where has the persistent infection occurred? Which genotype is involved? And can the healthcare system intervene before progression to cancer? In cervical cancer, vaccination, screening, triage, and treatment are linked within the most developed prevention pathway. For anal cancer, prevention is more strongly focused on high-risk groups and diagnostic capacity. In oropharyngeal cancer, HPV is important for understanding etiology and prognosis, but there is still no established population screening pathway. Evidence for vaginal, vulvar, and penile cancers must be interpreted separately by site.

## 1. Introduction

Infection with Human Papillomavirus (HPV) is very common, but the prevalence of infection does not mean that all infected individuals will develop cancer in the future. Most infections clear without specific treatment, and the main problem begins when a high-risk HPV genotype remains in the tissue for a long period. In these conditions, persistent infection can gradually induce cellular changes, and in some individuals, the progression pathway leads toward a precancerous lesion and eventually cancer. This pathway is best understood in cervical cancer. Using sensitive methods, HPV DNA is identified in almost all invasive cervical cancer samples and in over 95% of high-grade lesions close to cancer [1]. The disease usually begins with infection in the transformation zone and, if viral clearance does not occur, can progress from a precancerous lesion to invasive cancer. In this pathway, the persistence of high-risk genotypes, especially HPV16, is of great importance [2,3].

Cervical cancer therefore provides the most extensively characterized model for following HPV from infection through precursor disease to cancer. At this site, the natural history of infection is well studied, and intervention is possible at several stages of the disease pathway. Even here, however, not every positive result has the same meaning. HPV16 and HPV18 account for the largest share of cervical cancers, but other types such as HPV31, HPV33, HPV45, HPV52, and HPV58 may become significant depending on the population and histologic subtype. In multicenter studies, about half of cancers were related to HPV16 and nearly one-sixth to HPV18 [4]. Meanwhile, cancer risk is not uniform among high-risk genotypes, and HPV16 has shown the strongest association with risk in most studies [5]. Therefore, interpreting a positive test should not rely solely on the presence of the virus, but also on the genotype and individual conditions.

Examining cancers outside the cervix makes the differing roles of HPV in different anatomical sites clearer. In oropharyngeal cancer, the virus has been reported in about half of tumors, with HPV16 being the predominant type, although rates vary among regions and tumor subsites [6]. In oral cavity cancer, statistics are lower, and results are more dispersed, partly due to diagnostic methods and the lack of precise tumor site differentiation [7]. Even among HPV-positive tumors, not all patients have the same prognosis; some data suggest that non-HPV16 types may be associated with poorer survival. Therefore, determining the genotype becomes clinically significant before deciding on treatment de-intensification [8]. In anal cancer, the issue is different, as the risk is concentrated in people living with HIV, men who have sex with men (MSM), and some transplant recipients. Therefore, the logic of screening at this site relies more on high-risk groups [9], although there is still no standard global program for screening anal precancerous lesions [10].

Differences among anatomical sites are also seen in prevention strategies. In cervical cancer, programs have gradually moved from cytology-based screening to HPV-based screening and risk-based management. The start age for screening and screening intervals are adjusted based on the test type and individual risk [11,12,13]. The goal is to identify infections likely to cause high-grade lesions without subjecting many transient infections to unnecessary follow-up and intervention [14]. In this path, newer guidelines have accepted the use of self-collected samples and separate reporting of HPV16 and HPV18 [15,16]. Self-sampling and urine testing can make it easier for underscreened individuals to enter the program, but a positive result still requires clinical evaluation, triage, and follow-up [17,18,19].

Vaccination has shifted the point of intervention to the stage before persistent infection forms, whereas screening enters the care pathway only after an infection or tissue change has already occurred. Vaccinated cohorts are now entering screening, and evidence on screening frequency in these cohorts is developing [20]. This review examines how genotype, persistence, anatomical site, host status, assay interpretation, and access to prevention shape the clinical meaning of HPV detection. Its contribution is to connect these factors across sites and identify where detection can lead to a validated clinical action and where that link remains uncertain. This synthesis complements current cervical and anal guidelines [11,12,15,16,21] by considering the full pathway from vaccination through screening, triage, treatment, and follow-up. It does not derive new universal management thresholds or replace recommendations for a specific anatomical site.

## 2. Methods

### 2.1. Study Design

This structured narrative review was chosen to integrate genotype, persistence, anatomical context, and prevention pathways across clinically diverse HPV-associated cancers. The literature differed substantially in study design, population, anatomical site, specimen, assay, outcome definition, and follow-up, so evidence was synthesized qualitatively. No pooled effect estimates or formal statistical comparisons across anatomical sites were calculated. Numerical findings are reported within their original populations and methodological contexts and do not establish statistically significant differences between anatomical sites. Comparisons across sites describe biological interpretation, study characteristics, and prevention pathways. The absence of independent duplicate screening, formal risk of bias assessment, and a prospectively registered protocol limits reproducibility and means that the conclusions are interpretive rather than definitive.

### 2.2. Search and Selection of Sources

Identification of the literature was iterative and topic-specific, consistent with the structured narrative design of the review. PubMed, Scopus, and Web of Science were used as the principal bibliographic databases, while Google Scholar served only as a supplementary source for discovery and citation tracking rather than as a reproducible, exhaustive search engine. Searches had no publication-year restriction and were limited to English-language publications. Reference lists of relevant reviews, guidelines, and key empirical studies were also examined. Older foundational studies were retained when they directly informed natural history, genotype attribution, or longitudinal risk.

Search combinations linked an HPV concept with one or more thematic domains (anatomical site, genotype, persistence or clearance, biomarkers, screening or triage, vaccination, transmission, and prevention) and were adapted to each database and specific review question. Because the search was designed to support thematic coverage across heterogeneous anatomical sites and study designs rather than to generate a systematic-review denominator, full database-specific search strings and exact search dates were not archived in a reconstructible form.

Sources were eligible for inclusion when they addressed at least one predefined review domain: HPV genotype, infection persistence or clearance, anatomical site, natural history, biomarkers or molecular assays, screening or triage, vaccination, transmission, or prevention. For direct empirical synthesis, priority was given to original human studies and secondary analyses that reported relevant epidemiological, diagnostic, prognostic, longitudinal, screening, vaccination, or prevention outcomes. Sources were excluded from direct empirical synthesis when they were limited to laboratory or mechanistic findings without a clinically relevant human outcome, did not address one of the predefined review domains, or did not provide information relevant to the cross-site objectives of the review.

Reviews, meta-analyses, guidelines, consensus statements, and authoritative reports were eligible only as contextual sources. They were used to establish background, describe clinical or public-health context, identify relevant primary studies, and support interpretation, but their findings were not counted as independent empirical observations in the Results.

When multiple potentially relevant empirical sources addressed the same topic, priority was given to studies with direct human evidence, longitudinal or comparative designs where available, clearly defined outcomes, and sufficient methodological information to interpret the findings and the specific review theme. Independent duplicate screening was not performed, so there was no formal process for adjudicating disagreements between independent reviewers. Selection was based on relevance to the predefined themes and the priorities stated above.

### 2.3. Organization and Interpretation of Evidence

Evidence was organized qualitatively according to the main themes of the review. Interpretation took into account, where relevant, study design, population and sample size, representativeness, assay and specimen type, outcome ascertainment, control of confounding, follow-up duration, precision of estimates, and generalizability. These quantitative estimates were used to characterize the magnitude and precision of findings within their respective studies, not to generate direct statistical comparisons between anatomical sites. Differences in estimates were interpreted only in the context of study design, population characteristics, specimen type, assay methodology, outcome definition, and follow-up duration. Assays were not treated as interchangeable: HPV DNA detection indicates the presence of viral DNA; RNA or E6/E7 assays more closely reflect transcriptional activity; p16 is a surrogate marker in appropriate pathological contexts; Ki-67 and p16/Ki-67 dual staining reflect abnormal proliferation; methylation assays measure epigenetic alteration; and integration assays identify viral–host genomic events.

### 2.4. Methodological Considerations

A formal assessment of risk of bias using instruments appropriate to each study design and a formal certainty grading framework were not applied. Source selection was not performed through independent duplicate screening. The qualitative considerations listed in Section 2.3 support interpretation but do not replace formal appraisal or certainty grading. Restriction to English language publications may underrepresent regional evidence, particularly from settings with a high burden of HPV-associated disease. Its magnitude and direction cannot be estimated statistically because the literature in other languages was not assembled as a comparison set. The synthesis therefore cannot be regarded as an exhaustive global account of the evidence.

## 3. Results

### 3.1. Overview of Evidence by Site and Study Design

The evidence base encompassed randomized trials, prospective and retrospective cohorts, case–control and cross-sectional studies, tissue-based series, registry and population analyses, and secondary epidemiological analyses. Original human studies and secondary data analyses provided the direct evidence summarized in the Results, while reviews, meta-analyses, guidelines, consensus statements, and authoritative reports supported contextual interpretation and were not treated as independent empirical observations. The iterative narrative workflow did not generate a reproducible denominator or record-level attrition counts. The volume and methodological maturity of evidence differed across anatomical sites. The infection-to-persistence-to-precancer-to-cancer sequence was used as a conceptual framework primarily for cervical disease, where the natural history and precursor pathway are most extensively characterized. It was not assumed to represent a uniform biological sequence across all HPV-associated cancers. In particular, HPV-associated and HPV-independent pathways were considered separately where supported by the available site-specific evidence, and differences in precursor lesions, anatomical accessibility, diagnostic approaches, and natural history were retained in the site-specific synthesis. Cervical disease had the most extensively characterized evidence base for longitudinal natural history, precursor lesions, screening, and vaccination, whereas several non-cervical sites were represented mainly by smaller or more heterogeneous observational studies. Because HPV detection and biomarker assays measure different biological phenomena, quantitative findings were interpreted within the context of the assay, specimen type, sampling site, and outcome definition used in each study. Reported prevalence and prognostic estimates were therefore not treated as directly interchangeable across assays or anatomical sites. This difference reflects the structure and maturity of the available evidence rather than a formal statistical comparison of effect estimates between anatomical sites. Table 1 provides a structured evidence map by anatomical site and prevention pathway, while Table 2 distinguishes the principal genotypes, assays, and biomarkers used across the carcinogenesis and prevention continuum.

### 3.2. Cervical Cancer and Distribution of Predominant Genotypes

Cervical cancer is the clearest site for examining the association between genotype and cancer. In a multicenter study in France, viral DNA was identified in 501 out of 516 cases of invasive cancer. HPV16 was present in 72.9% and HPV18 in 18.8% of cases, with the share of HPV18 being higher in adenocarcinoma [22]. A similar pattern was seen in Mozambique, where HPV was identified in all cancer samples, and HPV16 and HPV18 together covered 71.7% of cancers. Importantly, some types such as HPV51 and HPV35 were more common in women without cancer. Therefore, the frequency of a genotype in the population does not necessarily mean a larger share in cancer [23]. In a large international study, eight genotypes—HPV16, HPV18, HPV31, HPV33, HPV35, HPV45, HPV52, and HPV58—together constituted 91% of HPV-positive cases, and HPV18 and HPV45 were more frequently seen in adenocarcinoma [24].

The strong association of HPV with cervical cancer has also been confirmed in case–control studies [25]. However, the rate of virus identification depends on sample quality and test sensitivity. Re-examination of samples initially reported as HPV-negative showed that part of the negative results was related to sample limitations or test methods, and with more sensitive methods, HPV identification in cervical cancer approached 100% [84]. Meanwhile, not all genotypes behave similarly. HPV16 remained longer in some studies, and in single-genotype infections, nearly half of high-grade lesions were associated with HPV16 [73,89]. Geographic differences are also observed. In Turkey, HPV16 was the most common type, whereas in Kenya, a more diverse pattern with the prevalence of HPV52, HPV16, and HPV67 was reported [75,76,77].

### 3.3. Persistence, Viral Load, and Lesion Progression

After determining the role of genotype, the more important question is how long the infection remains in the tissue. The difference between transient and persistent infection explains why HPV is very common, but cancer develops only in a small portion of individuals. In one study, women who were HPV-negative in the first two visits rarely developed lesions. In contrast, with persistence of HPV16 or HPV18 infection, the incidence of lesions rose from less than 1 per 1000 woman-months to nearly 9 per 1000 woman-months [49]. High and sustained viral load was also associated with the persistence of dysplasia [48]. Furthermore, the presence of viral DNA together with E6 and E7 transcripts was more strongly associated with subsequent persistence. In this context, RNA testing showed higher specificity for predicting subsequent dyskaryosis, although its sensitivity was lower [50].

Studies related to viral clearance show this pathway from another angle. Women who were negative from the start or cleared the infection did not develop high-grade lesions. In contrast, high viral load and persistent infection increased the risk of progression, and in some data, the cumulative incidence of lesions reached about one-third over 2 years [51]. With increasing age, the probability of viral clearance decreased, and higher viral load was more associated with persistence [80]. Most infections clear within 24 months, but the longer the infection persists, the more likely it is to continue in subsequent months [54]. In the placebo arm of a trial, HPV16 and HPV18 infections that persisted for at least 6 months showed a higher risk of lesion development [79].

Age and host factors also change the meaning of persistence. With increasing age, new infections decrease, but existing infections that have persisted become more important [81]. In a population-based cohort, persistent high-risk HPV infection, high viral load, and older age were all associated with a higher risk of progression [90]. Newer studies have also shown that six-month persistence of HPV16, HPV31, HPV52, and HPV58 is associated with high-grade lesions, although HPV16 still shows the strongest association [78]. In women undergoing surveillance for moderate lesions, HPV16 was associated with a higher probability of persistence or progression, especially when high-grade cytology was also present [82]. Even after cervical cancer surgery, the virus remains in some patients, and this persistence has been associated with menopause and surgical margin involvement [83].

Ciccarese et al. compared 292 individuals attending a sexually transmitted infection (STI) center with an apparently healthy population. Overall, genital HPV infection was detected in 50% of participants, including 51% of those attending the STI center and 43% of apparently healthy participants; this difference was not statistically significant (*p* > 0.05). Genital HPV infection was more prevalent among women than men (56% vs. 43%, *p* < 0.05). Among 48 HPV-positive participants retested after 12 months, type-specific HPV infection persisted in 33% (16/48), with higher persistence among women than men (52% vs. 16%, *p* < 0.05). Persistence was also more frequent among participants initially positive for high-risk HPV than among those with low-risk HPV (47% vs. 6%, *p* < 0.05) and among smokers than non-smokers (55% vs. 15%, *p* < 0.05). In multivariable logistic regression, smoking (OR, 6.89; *p* = 0.017) and high-risk HPV positivity (OR, 10.98; *p* = 0.044) remained significantly associated with persistence, whereas female sex and Ureaplasma parvum co-infection were no longer statistically associated with persistence. These findings support the importance of host and viral factors in HPV persistence, although the persistence analysis was based on a relatively small subgroup of HPV-positive participants followed for 12 months [91]. Across longitudinal cervical studies, persistent high-risk HPV was more strongly associated with progression than single-time-point HPV detection, although the magnitude of risk varied according to genotype, follow-up duration, study population, and assay.

### 3.4. Genotype and Magnitude of Risk

Genotype is not merely a type designation; it also modifies long-term risk. Among women with normal cytology, the 12-year risk of CIN3+ was 26.7% (95% CI, 21.1–31.8%) after HPV16 detection and 19.1% (95% CI, 10.4–27.3%) after HPV18 detection. Persistent HPV16 carried the highest risk, with 12-year CIN3+ risk reaching 47.4% (95% CI, 34.9–57.5%) [52]. In a 16-year follow-up study, cumulative cervical cancer risk was 13.5% (95% CI, 6.5–28.0%) among women positive for HPV16 and 10.3% among women positive for HPV58 without HPV16, compared with 0.26% (95% CI, 0.17–0.41%) among HPV-negative women [53]. These confidence intervals express statistical uncertainty within each study. The interval of 6.5% to 28.0% around the 16-year cancer risk is particularly wide and indicates limited precision. Between-study heterogeneity affects the comparability of estimates; it does not explain the confidence interval calculated within a single cohort. These estimates come from individual longitudinal studies and were not derived from a common cross-site analytical framework. Therefore, differences in absolute risk across studies should not be interpreted as direct statistical differences between anatomical sites. Readers should therefore interpret absolute risk differences with caution and avoid over-precision in cross-study comparisons.

Even within a genotype, virus behavior can differ. Some non-European variants of HPV16 have been associated with greater persistence, more severe lesions, and invasive cancer [92]. Regarding screening, HPV testing at the start of the study was able to separate long-term risk better than initial cytology. Specifying HPV16 added more information to risk stratification, and an HPV-negative result was associated with very low risk in subsequent years [93].

### 3.5. Host Environment, Microbiome, and Genetic Background

The fate of infection does not depend solely on viral characteristics. The mucosal environment and host characteristics can also change the probability of viral clearance or persistence. In a longitudinal study, the abundance of lactobacilli was associated with viral clearance, whereas Gardnerella and increased bacterial diversity were more commonly seen in individuals who developed persistent infection or high-grade lesions [88]. In women with HIV, persistent high-risk HPV infection remained common, and even after screening and treatment, some women remained HPV-positive or developed new lesions [94]. The genetic background of the host can also play a role. The HLA DRB1*13 allele has been associated with a higher probability of spontaneous regression of mild lesions, especially in the absence of HPV16 and HPV18 [87].

### 3.6. Oropharyngeal Cancer: From Exposure to Prognosis

In oropharyngeal cancer, HPV16 is the most important genotype, but the role of HPV at this site differs from the cervix. In a case–control study, HPV16 L1 seropositivity was strongly associated with oropharyngeal cancer (adjusted OR, 32.2; 95% CI, 14.6–71.3), whereas oral HPV16 infection was associated with an OR of 14.6 (95% CI, 6.3–36.6) [28]. HPV16 was the predominant genotype reported in HPV-positive oropharyngeal tumors in the cited studies [28]. Among patients with oropharyngeal cancer, those with HPV-positive tumors had a higher 3-year overall survival rate than those with HPV-negative tumors (82.4% vs. 57.1%; *p* < 0.001). After adjustment for age, race, tumor and nodal stage, tobacco exposure, and treatment assignment, HPV-positive status was associated with a 58% reduction in the risk of death (hazard ratio, 0.42; 95% CI, 0.27–0.66) [29]. These findings support the prognostic significance of tumor HPV status within the reported oropharyngeal cancer cohort but should not be interpreted as a pooled quantitative comparison with survival outcomes at other anatomical sites. In a study on patients and their spouses, nearly 90% of tumors were HPV-positive, but the prevalence of oral infection in spouses was much lower. Therefore, oral swabs mostly indicate the presence of oral infection and cannot replace tumor testing [30]. Data from Denmark and Mexico also showed the association of tumor HPV positivity with better survival and lower recurrence [31,32].

The value of HPV in oropharyngeal cancer is not only etiologic; it is also prognostic. In the cohort reported by Cao et al., 5-year overall survival among patients with HPV-positive disease was 89.3% (95% CI, 87.4–91.2%) [33]. Patients who remained free of disease progression for up to 24 months then had survival near that of their age-matched peers, whereas early disease progression was associated with a sharp decrease in survival. In some selected patients, treatment de-intensification after surgery was also associated with adequate survival, although the presence of extranodal extension still required careful risk assessment [95]. Among patients treated for recurrent HPV-positive oropharyngeal cancer, Kaplan–Meier estimates showed 2- and 5-year overall survival of 65% and 46% after solitary recurrence, compared with 19% and 9% after multisite recurrence (log-rank *p* < 0.001) [96]. Together, these studies support HPV status as an etiologic and prognostic tumor marker but do not establish an analogous population screening pathway.

### 3.7. Transmission, Sexual Behavior, and Oral Infection

To understand oropharyngeal cancer, transmission dynamics and oral HPV infection must also be considered, but this pathway is not simple or uniform. In the general population, a higher number of oral sexual partners and tobacco use were associated with oral infection, and even deep kissing has been proposed as one of the potential transmission pathways [97]. Men are more susceptible to oral infection and have slower viral clearance, and this difference may explain part of the higher prevalence of oropharyngeal cancer in men [98]. Population-based data have also shown that oral HPV16 is seen multiple times more in men than in women, and part of this difference is related to sexual behavior patterns [99]. However, in spouses of patients with HPV-positive oropharyngeal cancer, the prevalence of oral infection was similar to the general population. Therefore, HPV transmission among couples cannot be considered a linear and direct relationship [100].

Behavioral evidence and couple studies provide a more accurate picture of this pathway. A higher number of sexual partners, especially oral–genital partners, was more associated with oropharyngeal cancer than with other head and neck cancers, and HPV16 antibodies were also more common in these patients [101]. In a couple of studies, partner infection and genotype concordance were more strongly associated with oral HPV infection in men [102]. Genital transmission can occur in both directions, but for some oncogenic types, transmission from man to woman has been reported more often. Persistent infection was also associated with a higher probability of transmission and genotype concordance among couples [103]. On the other hand, the identification of HPV in some girls before their first vaginal intercourse shows that prevention should not be delayed until the start of full sexual activity, and vaccination should be done before the start of sexual contacts [104].

### 3.8. Anal Cancer and High-Risk Populations

Unlike the cervix, where screening logic has been formed at the population level, anal cancer risk is concentrated more in defined high-risk groups. In HIV-positive men who have sex with men, the prevalence of anal infection has been reported up to 95%, and in some studies, more than half of individuals had anal HSIL. This high disease burden has continued even after the expansion of antiretroviral therapy [34]. In longitudinal follow-up, HPV16 or HPV18 multiplied the risk of severe lesions, and a subgroup with low CD4 was identified, which would likely benefit more from screening [35]. In a screening program, abnormal cytology was seen in 60.4%, HPV in 92.6%, and oncogenic types in 79.5% of individuals. However, adding HPV testing to cytology did not significantly improve the diagnosis of anal HSIL and reduced the specificity of the test [36]. Therefore, in a population where infection prevalence is very high, HPV positivity alone does not have enough power to separate truly high-risk individuals.

Evidence from this site also shows an important operational point. The value of a test depends not only on its accuracy but also on what happens after a positive result. High-risk HPV was seen more in anal HSIL tissue than in normal tissue, and HPV16, HPV18, HPV52, and HPV58 were reported in these lesions [105]. p16 and Ki67 increased with higher lesion grade, E4 decreased, and methylation increased along the progression pathway to cancer, but the value of these biomarkers for predicting individual risk was still limited [86]. In men with HIV, anal infection and high-risk genotypes were much more common than in HIV-negative men [37]. With antiretroviral therapy initiated during acute HIV acquisition, baseline anal HSIL prevalence was similar to that reported in individuals without HIV, although subsequent anal HSIL incidence remained higher [39]. Structured screening programs have also shown that screening without regular follow-up is insufficient, and training the treatment team for the diagnosis and management of lesions is important [38,106]. Feasibility was also a limitation: in one anal screening study, 36 of 95 p16/Ki67 samples were unsatisfactory (37.9%; 95% CI, 28.1–48.4%) [38]. Overall, evidence at the anal site was concentrated in high-risk populations, and assay performance could not be separated from high-resolution anoscopy, treatment capacity, and follow-up.

Program outcomes illustrate the distinction between offering screening and completing subsequent care. In a Sydney pilot involving 136 people living with HIV, 64 of 69 referrals resulted in attendance for high-resolution anoscopy, 92.8% (95% CI, 83.9% to 97.6%). At 12 months, 37 of 52 participants due for repeat screening completed the required screening and referral steps, 71.2% (95% CI, 56.9% to 82.9%). These denominators describe different stages of the pathway, and the study could not estimate uptake among all invitees because invitation logs were incomplete [38]. In a Brazilian tertiary cancer center, an anal dysplasia service performed 324 examinations in 220 patients and delivered 69 treatments in 62 patients; two of three clinicians completed certification for high-resolution anoscopy. Long-term treatment effectiveness and cancer outcomes were not yet available [106]. These reports demonstrate feasibility and service delivery within selected settings, rather than a measured reduction in population anal cancer incidence.

### 3.9. Vaginal, Vulvar, and Penile Cancers

In vaginal and vulvar cancers, evidence is less than for cervical cancer, but the role of HPV in part of the disease is clearly visible. In a vaginal cancer study, viral DNA was identified in almost all squamous cell carcinomas, and HPV16 was the predominant type. HPV and p16-positive tumors also had better disease-specific survival [40]. In another study, HPV was seen in 75% of invasive vaginal cancers, and HPV-positive patients were diagnosed at a younger age [41]. In vaginal and vulvar lesions, genotypes covered by the 9-valent vaccine constituted a large part of HPV-positive cases, and HPV16 had a large share in high-grade lesions [42]. In vulvar cancer, the HPV-dependent pathway was mostly seen in high-grade lesions, whereas keratinizing carcinomas had a smaller share of HPV [43]. This difference shows that even in one anatomical site, not all tumors are necessarily created via the same pathway.

In penile cancer, the dispersion of results is higher. In one study, HPV was identified in 28.1% of samples, and HPV16 was the predominant type. p16 and E6 transcripts were also associated with HPV positivity [44]. Another study found high-risk HPV only in part of the tumors and showed no clear association with survival [45]. In contrast, another study reported HPV in nearly three-quarters of cases and identified HPV16, HPV31, and HPV35 as predominant genotypes [46]. This wide discrepancy shows that test method, tissue type, and study population composition can affect the estimate of HPV positivity. In precancerous lesions, HPV-dependent and HPV-independent pathways diverge, and HPV16 has a large share in the dependent pathway [47]. Across penile studies, prevalence estimates were therefore not directly comparable because specimen selection, tissue processing, and assay methodology differed.

### 3.10. Immune Status and Vulnerable Hosts

Immune system status can determine whether an infection clears or persists for a long time. In female kidney transplant recipients, the prevalence of anal high-risk HPV was much higher than in the control group. Cervical high-risk HPV acquisition was also higher, and the probability of viral clearance was lower [69,70]. In blood or bone marrow transplant recipients, the risk of some HPV-related squamous cell carcinomas, especially cervical and vulvar cancer, increased in long-term follow-up [71]. Women with HIV were also several times more exposed to cervical cancer than women without HIV, and a significant part of this burden was seen in sub-Saharan and East Africa [68]. In anal cancer, HIV also accounted for a significant part of the disease burden, especially in men [72].

### 3.11. HPV-Based Screening and Molecular Triage

In screening, the main goal is not just HPV detection, but separating individuals who are more likely to have or develop clinically significant lesions. In a large study, high-risk HPV was more common at younger ages, and its frequency increased with the severity of cytology. HPV16 and HPV18 also had higher predictive value than many other types [55]. Using the HPV result in first-round decision-making did not identify more high-grade lesions, but in the next round, the number of severe lesions was lower in the group whose HPV result was entered into management [56]. The importance of a negative result also became clear in long-term follow-up. The 6-year risk of CIN3+ after an HPV-negative result was less than half the risk after a cytology-negative result [57].

Data from low-resource countries show that the clinical value of screening is not limited to the test itself, and follow-up of positive results is also important. In Indian studies, test performance was not uniform across different centers, but a negative result was almost always associated with the absence of clinically significant disease [107]. In subsequent programs, the true effect of screening was better seen when HPV-positive individuals were referred for colposcopy [108]. One round of HPV testing, compared to usual care, reduced advanced cervical cancer and cervical cancer mortality [58]. In the next step, triage must separate those at higher risk among HPV-positive individuals. In the ATHENA analysis, an HPV16/18 plus dual-stain strategy detected 147 of 171 CIN3+ cases with 1012 colposcopy referrals, whereas the extended-genotyping strategy based on HPV16/18/31/33/45/52 detected 148 of 171 cases with 1238 referrals [73]. Viral integration has also been evaluated as a triage approach [85], but these assays measure different biological processes, and their comparative clinical utility depends on the population, specimen, and program setting.

### 3.12. Self-Sampling and Urine Testing

Self-sampling can improve access, but participation and subsequent clinical follow-up are separate outcomes. In a pragmatic randomized trial within an insured US health system, mailing HPV self-sampling kits increased screening completion among people overdue for screening from 18.8% with education alone to 35.7%, an absolute difference of 16.9 percentage points (95% CI, 13.8 to 20.0). Among those due for screening, completion was 61.7% versus 47.6%, a difference of 14.1 percentage points (95% CI, 11.2 to 16.9). Follow-up after a positive or unsatisfactory self-sample was completed by 46 of 54 participants in the due direct mail group and 20 of 23 in the overdue direct mail group. English-only materials and the insured population limit transfer to other settings [60].

In an organized screening pilot in Estonia, participation was 41.7% when kits were mailed directly, 34.1% when women were invited to request a kit, and 29.0% after a standard reminder. Both intervention groups could choose self-sampling or clinician sampling. The study did not establish completion of the additional examinations required after a positive self-sample [61]. These pragmatic results support improved participation, but they do not by themselves establish treatment completion or reduced cancer incidence. First void urine testing is another approach to specimen access; performance in the cited study was somewhat lower than for samples collected from the cervix in clinical settings [19].

### 3.13. Vaccination and Screening in the Vaccinated Generation

Vaccination evidence shows that intervening at the beginning of the infection pathway can prevent subsequent outcomes. The effect of the vaccine was not limited to reducing infection; a reduction in high-grade lesions and invasive cancer was also seen in population-based studies. In the HPV16 vaccine trial, efficacy against persistent HPV16 infection and related lesions was very high, and protection lasted for several years [62]. The bivalent vaccine also prevented persistent infection and high-grade lesions related to HPV16 and HPV18 in a trial in China [67]. In Sweden, quadrivalent vaccination was associated with a reduction in invasive cancer, and its effect was greater in individuals who received the vaccine before age 17 [63]. In England and Scotland, the greatest reduction in cancer and high-grade lesions was seen in individuals vaccinated at ages 12 to 13 [64,65,66].

The entry of vaccinated generations into screening age is the next stage of this pathway. In a study on women vaccinated against HPV16 and HPV18, a screening strategy with less frequent intervals did not increase high-grade lesions compared to a more frequent program [20]. As vaccine-type HPV became less prevalent in vaccinated populations, non-vaccine types represented a greater relative share of detected infections and lesions. Population-based data also showed a lower prevalence of vaccine-covered oral HPV types among vaccinated individuals than among unvaccinated individuals [109].

### 3.14. Global Burden and Distribution of HPV-Associated Cancers

To place these different pathways in an overall picture, the global disease burden must also be considered. The largest share of HPV-associated cancers still belongs to cervical cancer. In 2018, approximately 570,000 cases of cervical cancer and 311,000 deaths were estimated, with a large part of this burden in low-resource countries [26]. In the same year, approximately 690,000 new cancers were attributed to HPV, and nearly 80% of them were cervical cancers. However, oropharyngeal, anal, penile, vaginal, and vulvar cancers also had significant shares, and in men, a larger part of the disease burden was related to HPV16 and HPV18 [110]. Previous estimates had defined the share of HPV as about 4.5% of all cancers [111].

Newer data show another important point. A reduction in disease rates does not necessarily mean a decrease in the total number of cases. In 2022, approximately 662,000 cases of cervical cancer were reported, and head and neck cancers together constituted an important part of HPV-associated cancers. The disease burden was higher in countries with lower human development [27]. A 30-year analysis also showed that despite a reduction in age-standardized rates, the number of deaths from cervical cancer has increased [112]. In a GBD 2019 analysis of 49 Asian countries and territories, the number of incident cervical cancer cases increased from 153,146 in 1990 to 297,402 in 2019, representing a 1.94-fold increase, while the age-standardized incidence rate decreased from 12.73 to 11.70 per 100,000 [113]. Furthermore, the disease pattern is not identical in women and men. In the United States, increasing incidence of oropharyngeal and anal SCC was observed across several male racial and ethnic groups, supporting prevention strategies that are not focused exclusively on women [114].

## 4. Discussion

### 4.1. Identifying the Virus Is Not Equivalent to Cancer Risk

The most important finding of this review is that HPV positivity alone is not sufficient for risk stratification, because most infections are transient and clear without intervention. Risk increases when a high-risk genotype remains long-term in susceptible tissue or when the host has less ability for viral clearance. Therefore, to interpret risk, four components must be viewed together: genotype type, duration of infection persistence, involved tissue, and host immune status. The cervix provides the most extensively characterized setting for observing this pathway because evidence spans infection, precursor lesions, cancer, screening, and vaccination. This characterization refers to the breadth and maturity of the available evidence and does not imply a statistically demonstrated difference in HPV-related risk compared with other anatomical sites. Accordingly, the cervical pathway is used here as a reference framework rather than as a quantitative benchmark against which other anatomical sites are statistically compared.

The strength and clinical interpretability of the evidence differed across the main themes of this review. Evidence for cervical HPV persistence, genotype-specific risk, and HPV-based screening is supported by longitudinal studies and clinically relevant outcomes [48,49,50,51,52,53,57,58,78,90]. Tumor HPV status is also associated with prognosis in oropharyngeal cancer, although interpretation depends on the study population, treatment, smoking history, and outcome definition [29,31]. Evidence for anal screening and anal biomarkers is more dependent on the target population, sample adequacy, assay characteristics, and access to diagnostic evaluation [36,38,86]. Similarly, the practical value of self-sampling and urine testing depends on specimen type, assay performance, and completion of appropriate triage and follow-up [19,60,61]. These differences matter because a statistical association or improved test performance alone does not demonstrate improved clinical decisions or patient outcomes. The present synthesis therefore interprets effect estimates alongside study design, consistency, methodological characteristics, and clinical context. This approach distinguishes findings with more developed clinical evidence from those requiring further validation without assigning a formal certainty grade.

Findings from cervical disease require interpretation within each anatomical site because epithelial structure, precursor lesions, host immunity, and access to diagnostic evaluation differ. This review did not estimate the comparative contribution of local immune surveillance or hormonal influences across sites, so these factors cannot justify transferring cervical risk estimates or triage thresholds to other tissues. At the cervix, the transformation zone and identifiable precursor lesions support longitudinal studies of persistence and organized screening followed by colposcopy and treatment [2,3]. HPV-associated oropharyngeal cancer frequently arises in tonsillar tissue, where a comparable pathway from detectable infection to a screenable precursor lesion has not been established. Prognostic evidence concerns HPV status in tumor tissue, whereas HPV detection in oral samples has no established role in population cancer screening [2,28,29]. The anal canal also contains a squamocolumnar junction, but HIV-related immunosuppression and the burden of anal HSIL alter the clinical context. Anal screening therefore requires selection of appropriate risk groups and access to high-resolution anoscopy and treatment [9,21,34]. Vaginal cancer is represented by fewer and generally smaller studies, which limits inference about genotype-specific prognosis and prevention pathways [40,41]. Vulvar squamous carcinogenesis includes both HPV-associated and HPV-independent pathways, so cervical natural history cannot be applied to all vulvar lesions [43]. In penile disease, HPV attribution and biomarker interpretation vary with histological subtype and assay method; studies of invasive tumors and precursor lesions do not establish a population screening pathway equivalent to that of the cervix [44,47].

**Figure 1:**
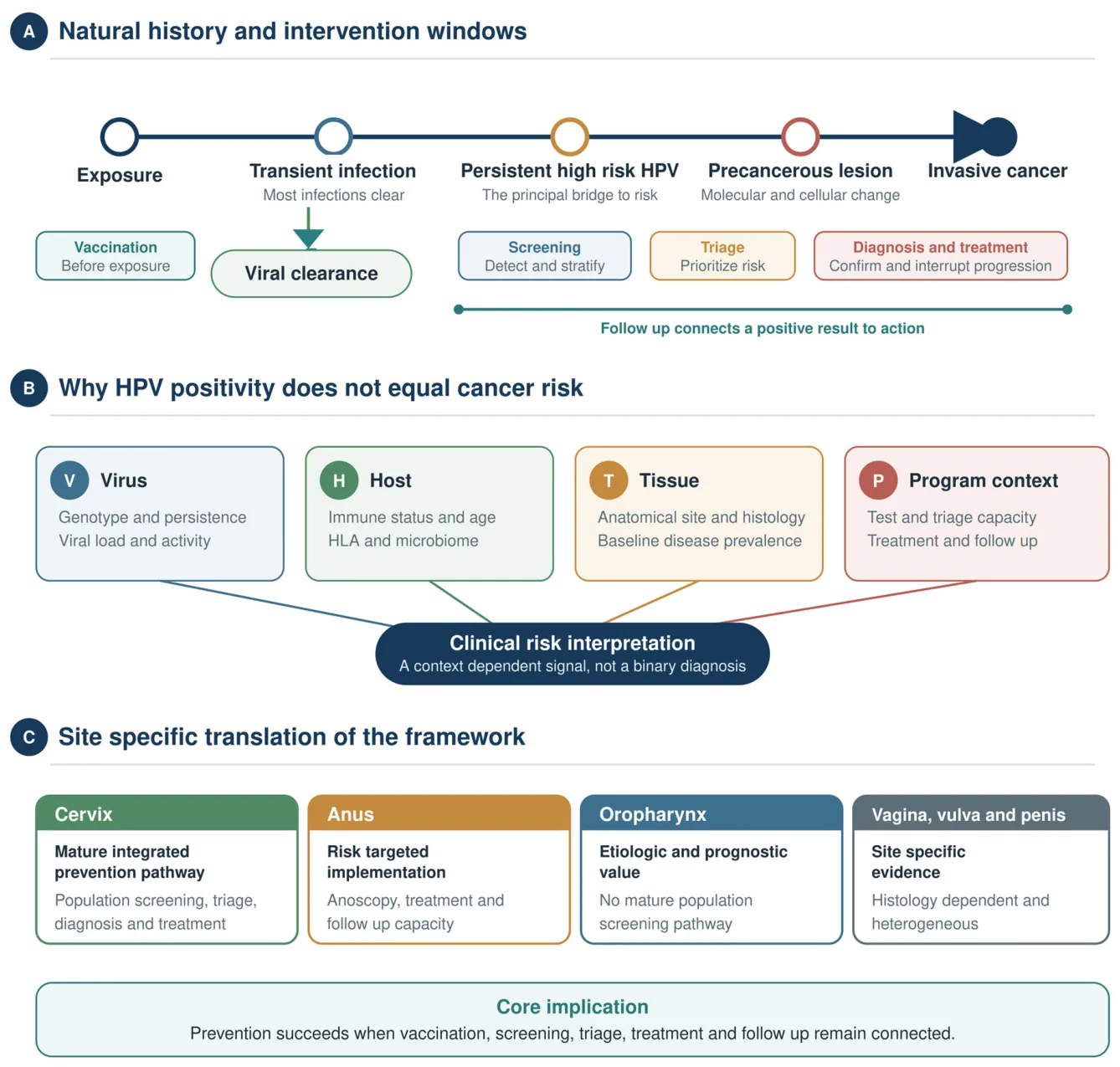
Contextual risk and prevention framework for HPV-associated cancers. The sequence from exposure through persistence, precancerous change, and invasive cancer is shown as a conceptual reference framework that is most extensively characterized in cervical disease and should not be assumed to occur identically across anatomical sites. The meaning of HPV positivity is shaped by viral, host, tissue, assay, and program factors, while biology, precursor pathways, screening feasibility, assay interpretation, and clinical implementation differ by site. The horizontal arrow shows possible disease progression. Colors distinguish different elements and do not indicate risk levels. HLA, human leukocyte antigen; HPV, human papillomavirus.

These differences limit the use of the cervical sequence of infection, persistence, precancer, and cancer to a conceptual reference framework. Clinical and public health implications therefore differ by site: cervical programs link population screening to treatment of precursors; anal prevention focuses on risk groups and specialized diagnostic capacity; oropharyngeal management uses tumor HPV status for prognosis and treatment planning; and vaginal, vulvar, and penile cancers require evidence specific to each site. Figure 1 integrates these dimensions with the natural history of infection, available opportunities for intervention, and the maturity of prevention pathways.

Assay heterogeneity is an important consideration when interpreting differences in reported HPV prevalence and prognostic associations across studies. HPV DNA PCR-based detection, RNA or E6/E7 transcript assays, p16/Ki-67 immunostaining, in-situ hybridization, methylation assays, and viral-integration analyses measure different aspects of HPV presence, biological activity, cellular response, or viral–host genomic alteration and therefore should not be treated as equivalent measures of HPV positivity. Differences in specimen type, sampling site, assay sensitivity and specificity, analytical thresholds, and the definition of HPV positivity may contribute to variation in reported prevalence, particularly in anatomical sites such as the oral cavity and penis. Similarly, prognostic associations based on tumor HPV DNA detection may not be directly comparable with those based on transcriptional activity, surrogate protein expression, or viral integration. Accordingly, the quantitative estimates summarized in this review were interpreted within the assay and specimen context of the original studies rather than pooled or treated as directly interchangeable measures of HPV-related disease.

### 4.2. Cervical Cancer as the Most Developed Prevention Benchmark

Cervical cancer has the most developed prevention pathway among HPV-associated cancers because intervention is possible at several stages of the disease’s natural history. Precancerous lesions are identifiable, there is a defined screening program, an HPV-negative result can indicate low risk for the coming years, and the vaccine effect has been confirmed in large studies. In recent years, guidelines have moved from cytology-based screening to HPV-based screening and risk-based management. In this approach, the start age and screening interval are adjusted based on the test result and the probability of high-grade lesion development [11,12,13]. Cytology examines cellular changes, while the HPV test identifies the presence of high-risk virus. Therefore, a suitable program must distinguish as much as possible between transient infections and cases more likely to progress [14].

This pathway continues to evolve with changing technology and the entry of vaccinated generations. The use of self-collected samples and separate reporting of HPV16 and HPV18 is an example of these changes [15,16]. An HPV-negative result usually gives more confidence about low risk in subsequent years, but a positive result does not always mean the presence of an important lesion and requires triage [2]. For this reason, the experience of cervical cancer shows an important principle: the value of a test is complete only when its result is connected to the next clinical decision. This experience offers a prevention benchmark for other HPV-associated cancers, but it cannot be transferred directly because precursor pathways, anatomical accessibility, screening feasibility, assay interpretation, disease prevalence, and implementation capacity differ by site.

### 4.3. Anal Cancer Requires Targeted and Risk-Based Implementation

In anal cancer, risk is concentrated more in defined high-risk groups. Anal infection and anal HSIL are more common in people living with HIV, men who have sex with men, and some transplant recipients. Therefore, screening for the entire population is not equally logical, and focusing on high-risk groups is more justified. However, selecting the population is only one part of the program. For screening to lead to cancer reduction, access to high-resolution anoscopy, lesion treatment, and long-term follow-up must also be available. Current recommendations focus more on identifying high-risk groups and selecting the appropriate time and threshold for referral, and there is still no single international protocol [9,21].

Anal cancer clearly demonstrates the difference between test accuracy and program effectiveness. The best test has a preventive effect only when an individual with a positive result enters additional evaluation and treatment. In cervical cancer, the colposcopy pathway and lesion treatment are more defined, but in anal cancer, limited access to anoscopy is one of the main problems. Self-sampling can make entering the testing stage easier, but it does not solve the follow-up and treatment problem on its own [10]. Therefore, increasing the number of tests without creating diagnostic and therapeutic capacity will likely have a limited effect on cancer reduction. At this site, program implementation is part of the prevention intervention itself, not a separate stage from it.

### 4.4. Oropharyngeal Cancer: Value of Etiology and Prognosis Without a Mature Screening Pathway

In oropharyngeal cancer, HPV is of great importance for understanding the cause of the disease and prognosis, but this importance has not yet turned into a suitable test for population screening. HPV16 is the predominant genotype, and patients with HPV-positive tumors usually have better survival. However, oral HPV infection is relatively common, and most infections never turn into cancer. For this reason, an oral swab cannot determine who will progress to cancer in the future. On the other hand, the rate of HPV positivity in oral cavity cancer studies varies greatly, and part of this difference is related to assay methods and the lack of precise tumor site differentiation [7]. This heterogeneity is also evident across oral lesions and oral squamous cell carcinoma. An Iranian systematic review and meta-analysis estimated an overall HPV prevalence of 21% (95% CI, 15–27%) across oral lesions and 31% (95% CI, 4–81%) in oral squamous cell carcinoma; heterogeneity was substantial (I^2^ = 84.5%, *p* < 0.001) [115]. The high I^2^ value indicates substantial between-study heterogeneity, suggesting that the pooled prevalence should be interpreted cautiously and that differences in population characteristics, anatomical definitions, specimen type, and HPV detection methods may contribute to the observed variability. Therefore, HPV prevalence at the population level and the presence of active HPV in tumors are two separate questions and should not be interpreted interchangeably.

This limitation does not diminish the importance of genotype stratification in a patient with a tumor. Some evidence suggests that tumors caused by non-HPV16 types may be associated with poorer survival and less p16 positivity. Therefore, before deciding on treatment de-intensification, HPV status and genotype must be carefully examined [8]. Currently, HPV at the tumor level is an important biomarker for disease etiology and prognosis, but at the population level, there is still no reliable screening test. For this reason, vaccination remains the most important primary prevention intervention for this site.

### 4.5. Vaginal, Vulvar, and Penile Cancers Must Be Interpreted Separately

Vaginal, vulvar, and penile cancers should be examined based on the specific evidence of each site. The share of HPV in each depends on tissue type, test method, and study population composition. In vulvar cancer, HPV-dependent lesions and HPV-independent cancers have two different pathways. In vaginal cancer, the virus has been seen in a large proportion of cases and has been associated with better survival in some studies, but the small number of patients limits the generalization of results. In penile cancer, the rate of HPV has also been reported to vary greatly from one study to another. Therefore, a single pooled estimate cannot adequately represent the role of HPV across these cancers, and prevalence estimates from different anatomical sites should not be directly compared without accounting for differences in specimen selection, tissue processing, assay methodology, population characteristics, and outcome definitions.

### 4.6. Persistence and Genotype Refine Risk Within Site-Specific Contexts

Persistence and genotype refine risk most clearly in cervical disease, where repeated type-specific detection has been studied longitudinally. Their interpretation elsewhere remains site-dependent because natural history, precursor pathways, specimen access, and assay meaning are not equally characterized. The meaning of a positive result changes with age, virus type, duration of infection, immune status, and tissue location. In young women, many infections are transient, whereas in older ages or when a high-risk type has persisted for a long time, that same positive result gains greater significance. HPV16 predominates in several HPV-associated cancers, but HPV18 plays a significant role in adenocarcinoma, and HPV35, HPV52, and HPV58 also have considerable shares in some regions [4]. Regional genotype patterns may also differ substantially. In the Middle East and North Africa, HPV16 and HPV18 remain the predominant high-risk genotypes, while other types show variable distributions across populations and countries [116].

### 4.7. Vulnerable Hosts Require Separate Prevention Logic

Individuals with immune deficiencies require different prevention programs because, in this group, it is not just about higher exposure rates, but also a lower probability of viral clearance. High-risk infections are more frequently seen in transplant recipients and people living with HIV, and some HPV-related cancers also have a higher prevalence. Reported excess risk varies across studies of different anatomical sites and immune-deficient populations, but these estimates should not be interpreted as directly comparable because the underlying populations, immune conditions, study designs, and outcome definitions differ [117]. This issue becomes more significant in anal cancer, because in some high-risk groups, the burden of lesions is high, but the capacity for high-resolution anoscopy is limited. Therefore, the start time, intervals, and intensity of screening must be adjusted based on the actual risk profile of the target population and the capacity of the healthcare system.

### 4.8. From Test Accuracy to Program Effectiveness: Triage, Self-Sampling, and Vaccination

Among people who test positive for HPV, clinically significant lesions are much less common than infection itself. Genotyping, dual staining, RNA assays, methylation, and integration testing have therefore been evaluated as approaches to refine risk stratification in selected settings. These measures are not interchangeable, performance differs by anatomical site and assay, and universal thresholds for routine clinical or cost-effective implementation across HPV-associated cancers have not been established [38,73]. Evidence for the performance of these biomarkers remains strongest in cervical screening, particularly from high-resource settings such as the ATHENA trial, where an HPV16/18 plus dual-stain strategy showed comparable detection of CIN3+ to extended genotyping with fewer colposcopy referrals. Data from low-resource environments are far more limited, and assay performance, sample adequacy, and downstream linkage to treatment cannot be assumed to transfer directly. The reviewed evidence likewise does not support routine universal p16/Ki67 implementation across anatomical sites, particularly given the substantial rate of inadequate samples reported in anal screening. Comparative diagnostic accuracy versus simpler approaches such as extended genotyping has been examined mainly in cervical populations; corresponding head-to-head data for oropharyngeal, anal, vaginal, vulvar, and penile sites remain sparse. Self-sampling may improve participation, but a positive result still requires timely diagnostic evaluation, treatment where indicated, and follow-up [10].

Regulatory status belongs to a named assay, specimen, intended use, and jurisdiction. In the United States, CINtec PLUS Cytology (Ventana Medical Systems, Inc., Tucson, AZ, USA) received FDA approval for specified cervical HPV screening and triage settings in 2020 [118], and the Aptima HPV assay (Hologic, Inc., San Diego, CA, USA) received approval for detection of E6/E7 mRNA from high-risk HPV in cervical specimens in 2011 [119]. Thus, viral RNA testing cannot be described as uniformly investigational. In Europe, the QIAsure FAM19A4 and miR124-2 methylation test (Self-screen B.V., Amsterdam, The Netherlands; distributed by QIAGEN, Hilden, Germany) has CE-marked in vitro diagnostic labeling for follow-up of positive HPV or ASC-US results, using specified cervical or vaginal self-collected specimens for cervical disease assessment [120]. CE marking, national guideline endorsement, and evidence of improved patient outcomes are distinct. These cervical indications do not authorize equivalent use for anal, oropharyngeal, vaginal, vulvar, or penile cancer screening; integration-based triage and applications of these markers at other sites in the studies summarized here still require validation of a clinical management pathway [38,85,86].

For cervical triage, the 2024 Enduring Consensus recommendations accept dual staining of HPV-positive results. Management uses established risk thresholds, including an immediate CIN3+ risk of at least 4% for colposcopy. Dual stain positivity generally leads to colposcopy and a negative result to repeat HPV-based testing in one year, while HPV16 or HPV18 positivity and high-grade cytology require their recommended management regardless of a negative dual stain result [12,121]. This is a threshold for a clinical action in a defined cervical setting, rather than a universal significance threshold or an economic decision rule for all anatomical sites.

Vaccination intervenes earlier in the natural history of the disease and acts before persistent infection forms. For this reason, performing it at younger ages shows greater effect. However, vaccination alone does not cover all components of prevention. Screening and vaccination coverage also remain unequally distributed across populations, particularly in high-burden and resource-limited settings. Socioeconomic disadvantage, lower education, rural residence, and poor access to healthcare have been identified as important contributors to these prevention gaps [122]. Vaccination coverage varies by country, vaccination history is not always fully and reliably recorded, and high-risk types outside vaccine coverage still exist in the population. Therefore, reducing the number or increasing the interval of screening must be done based on the individual’s actual risk and documented vaccination history, not just based on age or belonging to a vaccinated generation. Future programs should view vaccination and screening as components of a single, risk-based pathway.

Implementation barriers occur throughout the pathway. At invitation and participation, distance, costs, low health literacy, inaccurate contact details, and language barriers may limit uptake; outreach and a choice of sampling method can improve access [17,18,60,61,122]. At collection and testing, inadequate specimens, transport failures, unavailable reagents, and delays in communicating results can interrupt care, so programs need collection training and reliable laboratory and reporting systems [38,60,61,123]. After a positive result, shortages of colposcopy or anoscopy appointments, travel costs, and disconnected referral services can delay diagnostic evaluation [38,106,123]. At treatment and subsequent surveillance, limited trained staff, treatment capacity, and systems for recalling patients can prevent completion of care [38,106,123]. Program monitoring should therefore report participation, valid samples, receipt of results, completed diagnostic referral, indicated treatment, and retention separately.

Implementation context should change how a program is organized. A stronger health system can coordinate records, result communication, referral, and recall; settings with fragmented services should simplify visits and fund patient navigation before expanding testing [60,123]. Workforce capacity determines how many positive results can receive diagnostic evaluation and treatment, as the Brazilian program demonstrated through supervised anoscopy training [106]. Patient preferences influence whether individuals accept home sampling or a clinic examination, so offering an informed choice can improve participation [61]. Cultural acceptability depends on trust, privacy, understandable information, and engagement with local communities; the contextual implementation literature supports adapting delivery with community partners rather than assuming a mailed kit is acceptable to every group [17,18]. None of these adaptations replaces the need for follow-up after a positive result.

Biological or prognostic associations should be distinguished from improvements in clinical decisions or patient outcomes. In cervical screening, ATHENA data support p16/Ki67 dual staining as a triage approach and show that the number of colposcopy referrals can differ from extended genotyping [73]. These findings do not establish equivalent performance for all biomarkers or anatomical sites. Evidence for viral RNA, viral load, integration, and methylation depends on the specimen, assay threshold, population, and clinical endpoint, while studies of anal biomarkers remain more limited and heterogeneous [38,50,51,85,86]. Clinical adoption therefore requires evaluation of performance against an appropriate comparator, reproducibility, the consequences of referral, feasibility, and local resources. The evidence summarized here does not establish a single threshold or strategy that is clinically useful or cost-effective across HPV-associated cancers.

Economic evidence for dual staining is also specific to the setting. A US payer model of reflex dual staining after cervical cotesting projected incremental costs of US $15,231 (95% CI, US $10,717 to US $25,400) and US $23,487 (95% CI, US $15,745 to US $46,175) per quality-adjusted life year against cotesting with pooled HPV followed by reflex genotyping and cotesting with HPV genotyping without a reflex test, respectively [124]. A model informed by Kenyan screening data compared visual inspection with acetic acid alone with HPV testing followed by dual staining or visual inspection for triage. Dual stain triage became the preferred option above a modeled willingness to pay of 7115 international dollars per quality-adjusted life year; results were sensitive to test prices, visual inspection performance, and discounting [125]. These are model projections, with different comparators and costs, rather than observed trial evidence of economic benefit. The Kenyan willingness to pay value is not a universal threshold. Local evaluation should include specimen processing, laboratory and workforce capacity, diagnostic referral, treatment, losses to follow-up, and the program budget before a biomarker is adopted.

The ethical implications of screening based on individual risk also require consideration. HPV vaccination and screening coverage remain incomplete and uneven, particularly in settings with a high disease burden [122]. Reducing screening intensity in populations with incomplete vaccine protection could widen disparities if people facing socioeconomic disadvantage, lower education, rural residence, or poor access to healthcare are also less likely to be vaccinated or to complete follow-up after a positive result [122]. Program design should therefore combine risk-based management with prioritized outreach to underserved populations, simple referral pathways, and follow-up support. Programs should monitor vaccination and screening coverage, completion of diagnostic evaluation, and access to treatment by socioeconomic and geographic group [58,60,61,122]. These measures can help identify and address inequities as molecular triage, self-sampling, and individualized screening are introduced.

### 4.9. Limitations of Evidence

This review has limitations inherent to its narrative design. Evidence was synthesized qualitatively rather than through new pooled estimates. Because identification of the literature was iterative and topic-specific rather than prospectively logged as a systematic review, database-specific retrieval counts and record-level attrition were not generated within this workflow. Study characteristics were considered qualitatively as described in Section 2.3, but this did not constitute a formal assessment of risk of bias. Formal appraisal using instruments appropriate to each study design, certainty grading, and independent duplicate screening was not performed. Selection bias and incomplete reproducibility therefore remain limitations. Restriction to English language publications may underrepresent regional evidence, especially in settings with substantial HPV-related burden, although the magnitude of this bias cannot be quantified from the present evidence base. Direct cross-site comparison was further limited by heterogeneity in study design, population, anatomical site, specimen type, HPV detection and biomarker assays, outcome definitions, and follow-up, together with unequal methodological maturity across sites. Biomarker performance and implementation evidence continue to evolve. These limitations do not remove the value of a narrative approach for integrating clinically diverse evidence, but they require cautious interpretation of cross-site comparisons and of the distinction between biological association and demonstrated clinical utility.

### 4.10. Research Priorities and Interpretive Summary

Future research should evaluate the consequences of HPV detection for clinical decisions and patient outcomes. Evidence on less frequent cervical screening in vaccinated cohorts supports further follow-up of appropriate intervals and implementation under routine conditions [20]. Studies involving men and cancers outside the cervix need larger populations and standardized specimen and assay definitions to address the variability illustrated by the penile and anal literature [38,44,45,46,47]. Anal program evaluation should measure access to anoscopy, indicated treatment, and retention, alongside test accuracy [21,38,106]. Regional surveillance should track HPV types beyond HPV16 because genotype distributions and risks differ [4,5,116]. Comparative studies of genotyping, dual staining, RNA, methylation, and integration should evaluate referral burden and clinical outcomes, while economic analyses should test assumptions about costs, service capacity, and follow-up in the intended setting [73,85,86,121,124,125].

The cervical natural history provides a conceptual reference because persistence and identifiable precursor disease can be connected to screening and treatment [2,3,52,58]. At other sites, HPV attribution, precursor pathways, assay interpretation, and access to diagnostic evaluation differ [28,34,40,43,44,47]. Vaccination can prevent targeted infections and reduce subsequent cervical cancer risk [62,63,64,65,66,67], while screening and triage require an organized response to positive results and continued follow-up [12,38,60,61,123]. This review connects those findings across sites to explain where HPV detection informs a clinical action and where the prevention pathway remains incomplete. It complements recommendations for specific sites and does not replace their management thresholds [11,12,21,121].

## 5. Conclusions

HPV-related cancers cannot be interpreted solely based on a positive or negative result. Risk interpretation should therefore integrate genotype, persistence, anatomical context, host status, and the biological meaning of the assay used. Cervical cancer provides the most extensively characterized prevention model because vaccination, screening, triage, and treatment are linked within a defined pathway. This model serves as a reference for understanding prevention pathways, but the available evidence does not support direct statistical ranking of HPV-associated cancer sites because study populations, outcomes, assays, and follow-up differ substantially across sites. In oropharyngeal cancer, HPV is important for etiology and prognosis, but there is still no reliable pathway for population screening. Vaginal, vulvar, and penile cancers must also be interpreted based on site-specific evidence. Ultimately, the success of prevention is not limited to the number of tests. Vaccination, screening, triage, treatment, and follow-up must become a continuous, risk-based pathway so that virus identification truly leads to cancer reduction.

## Figures and Tables

**Table 1:** Structured evidence map by anatomical site and prevention pathway in HPV-associated cancers.

Site or Pathway	Principal Message of the Evidence	Representative Empirical Sources
Cervical cancer	Persistent high-risk HPV is closely linked to precancerous lesions and invasive disease; the evidence base for longitudinal natural history and screening is more extensively characterized at this site than at several non-cervical sites.	[22,23,24,25,26,27]
Oropharyngeal cancer	HPV16 has strong etiologic and prognostic importance, but no established population precursor-screening pathway analogous to cervical screening exists.	[28,29,30,31,32,33]
Anal cancer	HPV and high-grade lesions are frequent in high-risk groups, but prevention requires anoscopy, lesion treatment, and sustained follow-up rather than detection alone.	[34,35,36,37,38,39]
Vaginal and vulvar cancers	Vaginal disease is rare and supported by a limited evidence base; vulvar disease includes HPV-associated and HPV-independent pathways.	[40,41,42,43]
Penile cancer	Estimates vary across heterogeneous tissue-based studies and depend on sampling and assay methodology; HPV16 predominates among HPV-positive cases.	[44,45,46,47]
Persistent high-risk infection	Persistent high-risk infection is a major determinant of progression risk, particularly in cervical disease, although its role and clinical interpretation vary by anatomical site.	[48,49,50,51,52,53,54]
HPV-based screening	A negative result indicates very low short-term risk, while a positive result becomes clinically useful only when linked to triage.	[55,56,57,58,59]
Self-sampling and urine testing	These approaches lower the barrier of clinic-based sampling and raise participation, but they remain dependent on triage and follow-up.	[19,60,61]
Vaccination before exposure	Vaccination before exposure reduces persistent vaccine-type infection, CIN3, and invasive cancer, with stronger effects at younger ages.	[62,63,64,65,66,67]
The vulnerable host	HIV, kidney transplantation, and blood or marrow transplantation shift HPV toward persistence, recurrence, and higher risk, calling for a separate logic of prevention.	[68,69,70,71,72]

**Table 2:** Genotypes and biomarkers along the HPV carcinogenesis and prevention pathway.

Category	Biomarker or Genotype	Clinical Meaning	Sources
HPV16	HPV16	The most prevalent and stable high-risk genotype across multiple anatomical sites and strongly associated with persistent infection and a wide range of HPV-positive cancers.	[22,24,29,31,52,53,73,74]
HPV18	HPV18	One of the two most clinically important genotypes in cervical cancer; it accounts for a larger proportion of adenocarcinomas.	[22,23,24,67]
Other high-risk genotypes	HPV31, HPV33, HPV35, HPV45, HPV52, HPV58	These genotypes contribute significantly in certain regions and histological subtypes.	[23,24,40,75,76,77,78]
Persistent high-risk infection	Persistent high-risk infection	Repeated detection of the same genotype over time is a more important predictor of disease progression than a single positive test.	[49,79,80,81,82,83]
Viral detection, activity, and integration	HPV DNA, HPV RNA/E6/E7, viral integration	HPV DNA detection indicates the presence of viral DNA; RNA or E6/E7 assays more closely reflect transcriptional activity; and integration assays detect viral–host genomic events. Results are not directly interchangeable.	[28,50,59,84,85]
p16, Ki67, and methylation	p16, Ki-67, and methylation biomarkers	p16 is a surrogate marker in appropriate pathological contexts, Ki67 and p16/Ki67 dual staining reflect abnormal proliferation, and methylation assays measure epigenetic alteration. Their predictive and clinical utility is not uniform across anatomical sites.	[30,31,38,86]
Molecular triage	Molecular genotyping and targeted tests	Partial genotyping and targeted molecular tests have been evaluated to refine risk stratification among HPV-positive individuals, but comparative clinical utility and implementation thresholds vary by assay, population, and anatomical setting.	[59,73,85]
Microbiome and HLA	Vaginal microbiome and HLA	The vaginal microbiome and certain host genetic factors, including HLA, may influence viral clearance, persistence, or lesion regression.	[87,88]

## Data Availability

No new data were created or analyzed in this study. Data sharing is not applicable to this article.
